# Myeloid masquerade: Microglial transcriptional signatures in retinal development and disease

**DOI:** 10.3389/fncel.2023.1106547

**Published:** 2023-01-26

**Authors:** Kristen M. Pitts, Milica A. Margeta

**Affiliations:** ^1^Department of Ophthalmology, Massachusetts Eye and Ear, Harvard Medical School, Boston, MA, United States; ^2^Schepens Eye Research Institute of Mass, Eye and Ear, Boston, MA, United States

**Keywords:** microglia, monocytes, retina, retinal development, neurodegeneration, single cell RNA sequencing

## Abstract

Microglia are dynamic guardians of neural tissue and the resident immune cells of the central nervous system (CNS). The disease-associated microglial signature (DAM), also known as the microglial neurodegenerative phenotype (MGnD), has gained significant attention in recent years as a fundamental microglial response common to various neurodegenerative disease pathologies. Interestingly, this signature shares many features in common with developmental microglia, suggesting the existence of recycled gene programs which play a role both in early neural circuit formation as well as in response to aging and disease. In addition, recent advances in single cell RNA sequencing have revealed significant heterogeneity within the original DAM signature, with contributions from both yolk sac-derived microglia as well as bone marrow-derived macrophages. In this review, we examine the role of the DAM signature in retinal development and disease, highlighting crosstalk between resident microglia and infiltrating monocytes which may critically contribute to the underlying mechanisms of age-related neurodegeneration.

## Introduction

Microglia, the resident immune cells of the central nervous system (CNS), have emerged as essential players in the development and degeneration of neural parenchyma (Li and Barres, 2018). They are a yolk sac-derived myeloid lineage distinct from bone marrow and fetal liver monocytes and, under physiological conditions, are long-lived and self-renewing without contribution from the peripheral immune system (Alliot et al., 1999; Ajami et al., 2007; Ginhoux et al., 2010; O’Koren et al., 2016; Askew et al., 2017; Reu et al., 2017; Tay et al., 2017). Microglia are exquisitely complex and dynamic cells, surveilling the entirety of neural parenchyma every few hours and exhibiting high sensitivity to even subtle changes in their microenvironment (Nimmerjahn et al., 2005; Hickman et al., 2013; Gosselin et al., 2017). Historically, microglia have been categorized as either “resting” or “activated” based largely on morphology and the presence of certain cell-surface markers (Butovsky and Weiner, 2018). Among activated microglia, a distinction has been drawn between proinflammatory “M1” (classically activated) or anti-inflammatory “M2” (alternatively activated) microglia (Colonna and Butovsky, 2017). New approaches in RNA sequencing, however, have revealed the need for more granular analyses of microglial phenotypes and functions.

In the context of aging and neurodegeneration, microglia acquire a unique transcriptional signature characterized by up-regulation of proinflammatory, phagocytic, and lipid metabolism genes (Keren-Shaul et al., 2017; Krasemann et al., 2017; Hammond et al., 2019). This signature, referred to as the disease-associated microglial (DAM) phenotype, has been associated with several models of CNS degeneration and is distinct from microglial activation associated with lipopolysaccharide stimulation or viral infection (Holtman et al., 2015; Friedman et al., 2018). Although the term microglial neurodegenerative phenotype (MGnD) may also be used in these contexts, we will predominately refer to “DAM” in this review in alignment with cited literature. Intriguingly, several genes characteristic of the DAM phenotype are up-regulated by postnatal microglia during periods of white matter refinement, cell death, and synaptic pruning (Butovsky et al., 2014; Hagemeyer et al., 2017; Wlodarczyk et al., 2017; Anderson et al., 2019a,^2022^; Hammond et al., 2019; Li et al., 2019), as well as by retinal microglia in response to certain regeneration paradigms (Todd et al., 2020). Developmental remodeling is a finely orchestrated process, with dysregulation of microglial reactivity leading to impaired synaptic circuits and the onset of neurodevelopmental disorders (Paolicelli et al., 2011; Sellgren et al., 2017; Carloni et al., 2021; Xiao et al., 2021). Thus, how DAM microglia promote normal tissue maturation in development but are associated with pathological neuron loss in disease remains an open area of investigation.

In this review, we examine the role of the DAM signature in retinal development and retinal disease, identifying cell death and phagocytosis of myelin components as unifying stimuli. We then highlight recent computational advancements which have revealed significant and previously unseen differences between *bona fide* DAMs and infiltrating disease-inflammatory macrophages (DIMs) in the brain, the latter of which are virtually absent in development but increase with aging (Silvin et al., 2022). Finally, we propose a speculative model in which the interplay between microglia and DIMs in disease contributes to a state of maladaptive reactivity, leading to chronic inflammation and neurodegeneration.

### Disease-associated microglia (DAM)

The DAM phenotype was first identified by single cell RNA sequencing of isolated myeloid cells in a transgenic Alzheimer’s disease (AD) model, revealing a core microglial signature which is conserved in human disease (Keren-Shaul et al., 2017). This microglial transcriptional profile is characterized by acquisition of CD11c (Itgax), a leukocyte-activating and complement-associated integrin (Benmamar-Badel et al., 2020), as well as a suite of phagocytic and lipid metabolism genes proposed to function in the clearance of amyloid beta (Aβ) plaques. In both mouse and human disease, DAM microglia were spatially located around plaques, with phagocytes in these regions exhibiting nearly complete co-expression with lipoprotein lipase (LpL), a key metabolic enzyme involved in the clearance of myelin lipid debris (Eckel and Robbins, 1984; Bruce et al., 2018). Interestingly, acquisition of the DAM phenotype was shown to be biphasic and dependent in part on Triggering Receptor on Myeloid Cells 2 (Trem2), a well-established genetic risk factor for AD (Gratuze et al., 2018). In Stage 1 DAM, microglia up-regulated apolipoprotein E (ApoE) with concurrent down-regulation of the microglial homeostatic program (e.g., *Cx3cr1*, *P2ry12*, and *Tmem119*). This Trem2-independent stage led successively to a Trem2-dependent stage, Stage 2 DAM, characterized by full acquisition of the DAM signature (e.g., *Itgax*, *LpL*, *Spp1*, and *Clec7a*).

Another foundational report published the same year as Keren-Shaul et al. (2017) demonstrated the existence of a disease-associated cluster in mouse models of AD, amyotrophic lateral sclerosis (ALS), and multiple sclerosis (MS) using bulk RNA sequencing of isolated microglia (Krasemann et al., 2017). This transcriptional profile, termed the MGnD, highlighted several key genes shared with the DAM signature (e.g., *ApoE*, *Trem2*, *Spp1*, *Clec7a*) with increased attention to proinflammatory mediators such as *Ccl2*. Krasemann et al. (2017) further demonstrated that DAM microglia are spatially localized around Aβ plaques in AD and that ApoE expression is positively correlated with severity of disease in mouse models of MS and ALS. Of significance, this report showed that up-regulation of MGnD genes could be elicited in microglia *via* stereotactic administration of apoptotic neurons, indicating that the presence of dead neurons is sufficient to induce the MGnD microglial phenotype. Activation of MGnD in response to transplanted dead neurons was critically dependent on ApoE, such that ApoE knockout (KO) mice exhibited suppression of key disease-associated markers, including the secreted lectin Galectin-3 (*Lgals3*). Mechanisms of MGnD activation in response to apoptotic neurons may include several cell-death cues, including phosphatidylserine exposure and increases in extracellular ATP (Inoue, 2002; Scott-Hewitt et al., 2020; Park et al., 2021; Kurematsu et al., 2022; Ma et al., 2022).

### DAM in white matter development and degeneration

A reciprocal relationship between microglial *ApoE* and homeostatic gene expression has also been demonstrated in neurodevelopment (Butovsky et al., 2014; Hammond et al., 2019; Li et al., 2019), suggesting that postnatal microglia may encounter similar challenges in their microenvironment, including neuronal apoptosis and myelin debris clearance. Indeed, it has been estimated that half of the postnatal CNS cell population must be eliminated and cleared early in development (Oppenheim, 1981; Burek and Oppenheim, 1999), placing significant burden on microglial functions. Developmental microglia thus constitute an exceedingly heterogenous population (Hammond et al., 2019; Li et al., 2019), which are responsible for performing diverse roles in the postnatal brain and retina, including synapse formation (Paolicelli et al., 2011; Parkhurst et al., 2013; Miyamoto et al., 2016; Weinhard et al., 2018), modulation of axonal growth (Pont-Lezica et al., 2014; Squarzoni et al., 2014), secretion of key trophic factors (Ueno et al., 2013), and clearance of redundant neuronal precursors (Cunningham et al., 2013). Single cell RNA sequencing of brain microglia across the murine lifespan revealed a developmental peak in the DAM signature at P5 (Hammond et al., 2019); however, the functional significance of this peak is not completely understood.

One of the strongest overlaps between disease-associated and developmental microglia exists between DAM microglia and proliferative-region associated microglia (PAM), a subset of CD11c^+^ developmental microglia characterized by ameboid morphology, high metabolic activity, and up-regulation of genes such as *ApoE*, *LpL*, *Spp1*, and *Clec7a* (Li et al., 2019). The identification of PAM builds upon a prior body of work identifying a sharp increase in CD11c^+^ brain microglia between P3 and P5, which begins to decline significantly by P7 (Wlodarczyk et al., 2017). The emergence of the PAM microglial population coincides with a wave of programmed oligodendrocyte death during early myelination, suggesting that this subset may play a role in efferocytosis and metabolism. Interestingly, the appearance of PAM is independent of ApoE and Trem2 (Li et al., 2019). Common to both the DAM and PAM signatures is expression of insulin growth like factor 1 (Igf1), a neurotrophic factor involved in neurogenesis (Nieto-Estevez et al., 2016) and oligodendrocyte precursor cell (OPC) survival (Hagemeyer et al., 2017; Wlodarczyk et al., 2017), suggesting that DAM-like microglia in this context may simultaneously mediate both cell elimination and cell survival. Indeed, it has been demonstrated that microglia engulf both apoptotic and non-apoptotic OPCs in the corpus callosum between P4 and P11 and are thus active modulators of white matter development (Nemes-Baran et al., 2020). A similar transcriptional profile has been reported for a subset of axon tract-associated microglia (ATM), which occupy regions adjacent to heavily myelinated axons prior to myelination onset (Hammond et al., 2019). These subsets reflect significant heterogeneity in microglial states and functions during early CNS development.

A diversity of microglial states has also been observed in the context of white matter aging. In contrast to the Trem2-independent formation of PAM microglia, which facilitate the phagocytosis of oligodendrocytes and OPCs during white matter development (Li et al., 2019), a Trem2-dependent subset of white matter-associated microglia (WAM) have been described in the context of aging and AD (Safaiyan et al., 2021). This microglial subset has been shown to play a key role in the uptake of myelin debris and shares several features of both PAM and DAM microglia, including down-regulation of the homeostatic microglial program with strong up-regulation of disease-associated genes (Safaiyan et al., 2021). One of the most strongly up-regulated genes by WAM microglia was *Lgals3*, the gene encoding the carbohydrate-binding lectin Galectin-3, which has previously been shown to facilitate myelin debris clearance by primary microglia *in vitro* (Rotshenker et al., 2008). Prior work has demonstrated that Trem2 may bind anionic lipid species (Wang Y. et al., 2015; Ulrich et al., 2017) including various phospholipids (Cannon et al., 2012) and act as a receptor for myelin debris uptake (Cantoni et al., 2015; Poliani et al., 2015; Wang Y. et al., 2015); however, Safaiyan et al. (2021) demonstrated that Trem2 is not required for microglial engulfment of myelin basic protein despite its critical role in promoting lysosomal activity and initiating the WAM signature. This finding implicates Trem2 in the control of downstream genetic programs and points toward the presence of compensatory lipid-sensing receptors on the microglial surface which may aid in myelin phagocytosis.

In addition to its accumulation during aging and age-related disease, myelin debris may be generated as the result of traumatic CNS injury (Kopper and Gensel, 2018). Following spinal cord injury, it has been demonstrated that myelin debris inhibits axonal regeneration (McKerracher et al., 1994), remyelination, and oligodendrocyte differentiation (Kotter et al., 2006), while acting as an inflammatory stimulus to local macrophages (Kroner et al., 2014; Wang X. et al., 2015). Thus, the physiological clearance of myelin debris by recruited microglia and macrophages may serve to promote a pro-regenerative CNS environment (Neumann et al., 2009). The phagocytosis of opsonized myelin is facilitated by complement-mediated inflammatory pathways – such as those downstream of complement receptor 3 (CR3) –which can lead to the activation of FAK/PI3K/Akt/NF-κβ signaling and increased proinflammatory cytokine production (Sun et al., 2010). Conversely, activation of Trem2 pathways has been shown to lead to anti-inflammatory clearance of myelin debris in experimental autoimmune encephalomyelitis (EAE) (Takahashi et al., 2007); however, whether this receptor plays an anti-inflammatory role following acute nerve injury is not known. Following optic nerve crush (ONC) injury, a model of optic neuropathy leading to retinal ganglion cell (RGC) degeneration, it has been demonstrated that complement proteins C1q, C3, and CR3 are necessary for RGC regeneration (Peterson et al., 2021). It was shown that C1q opsonizes myelin debris for clearance by CR3^+^ microglia, a mechanism which reflects the reparative activity of macrophages following peripheral nerve damage (Barrette et al., 2008). Taken together, these studies implicate a critical role for microglia and macrophages in managing degenerated myelin components and promoting CNS repair.

### DAM in retinal development

**FIGURE 1:**
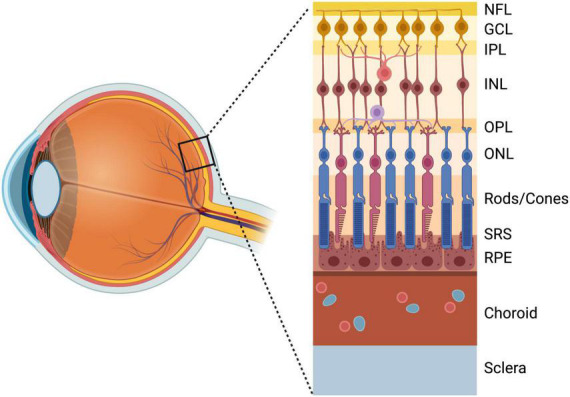
Anatomy of the retina anterior to posterior: NFL, nerve fiber layer; GCL, ganglion cell layer; IPL, inner plexiform layer; INL, inner nuclear layer; OPL, outer plexiform layer; ONL, outer nuclear layer; Photoreceptor layer (Rods/Cones); SRS, subretinal space; RPE, retinal pigment epithelium; Choroid; Sclera. Created with www.biorender.com.

Although microglia exhibit regional heterogeneity in distinct CNS compartments (De Biase et al., 2017; O’Koren et al., 2019), it has been demonstrated that retinal microglia are ontogenetically similar to microglia of the brain and spinal cord (Silverman and Wong, 2018; O’Koren et al., 2019). These cells colonize the retinal parenchyma prior to E11.5, transiently expressing markers of activation, including CD45 and CD68 (Hume et al., 1983; Santos et al., 2008; Sierra et al., 2014). In adulthood, retinal microglia are laminarly distributed in the inner plexiform and outer plexiform layers, with small numbers also present in the nerve fiber layer (NFL) and ganglion cell layer (GCL), but are essentially absent from outer retinal layers (Silverman and Wong, 2018; Figure 1). As in the brain, microglia colonize the retina in pursuit of neuronal “eat-me” signals (Medina and Ravichandran, 2016; Silverman and Wong, 2018) a concerted process which may be disrupted by inhibition of programmed cell death (Casano et al., 2016; Xu et al., 2016). After entry into the retina, it has been shown that microglia refine retinal circuitry by both removing dead and dying “corpses” (Brown and Neher, 2014; Reichenbach and Bringmann, 2016; Anderson et al., 2022) and contributing to pro-death processes *via* secretion of toxic factors and proinflammatory cytokines, including tumor necrosis factor α (Tnf-α) (Sedel et al., 2004). Recently, it has been demonstrated that microglia phagocytose non-apoptotic RGCs *via* C1q-CR3 signaling (Anderson et al., 2019b), although the mechanisms which direct this fatal “tagging” by complement remain poorly understood.

Using bulk RNA sequencing of retinal microglia in development, Anderson et al. (2019a) have demonstrated that retinal microglia acquire a CD11c^+^ signature with peak postnatal density at P7. Isolated CD11c^+^ retinal microglia also shared marked similarities with both DAM and PAM microglia, including up-regulation of *ApoE*, *Spp1*, *Clec7a*, and *Igf1*, with concurrent down-regulation of *Cx3cr1* and other microglial homeostatic genes. Similar to the developmental PAM subset (Li et al., 2019), CD11c^+^ retinal microglia were shown to be largely ApoE- and Trem2- independent, such that ApoE KO resulted in down-regulation of *Itgax*, while Trem2 KO resulted in down-regulation of *Itgax*, *LpL*, and *Cd68*, with selective up-regulation of *Tmem119*. Other DAM genes remained unaffected, including *ApoE* levels in Trem2 KO retinas, and the total number of DAM-like microglia remained unchanged. Although the downstream effect of genetic targeting of *ApoE* and *Trem2* in developmental retinal microglia was modest in comparison to the ablation of these genes in disease, the effect of these KOs on microglial function and postnatal RGC density remains an open question.

In the absence of the pro-apoptotic factor Bax (Pequignot et al., 2003), postnatal retinal microglia retained a predominately homeostatic signature, suggesting that it is the presence of apoptotic neurons in development which drives acquisition of the DAM-like program (Anderson et al., 2019a). In a subsequent report, single cell RNA sequencing revealed distinct microglial subclusters in the postnatal retina which were similarly dependent on Bax (Anderson et al., 2022). Retinas harvested from Bax KO mice exhibited a five-fold increase in the homeostatic microglial cluster (e.g., *P2ry12*, *Tmem119*) compared to wild-type, with concurrent decreases in the ApoE-enriched remodeling cluster (e.g., *ApoE*, *Ctsb*), chemokine/cytokine expressing cluster (e.g., *Cxcl2*, *IL-1*β), ATM-PAM-like cluster (e.g., *Spp1*, *Fabp5*), and PLX-enriched cluster (e.g., *Npl*, *Apoc1*). This report further demonstrated that CR3 and Mer receptor tyrosine kinase (MerTK) are critical for microglia-mediated efferocytosis of RGCs, while Axl receptor tyrosine kinase is dispensable for RGC clearance but essential for mediating microglial Csf1r-independence (Anderson et al., 2022). Activated CD11c^+^ microglia in the developing retina were shown to be Csf1r-independent, such that they resisted depletion with the Csf1r inhibitor PLX3397 and subsequently comprised significantly higher proportions of the retinal microglial population (Anderson et al., 2019a,^2022^). Interestingly, Csf1r-independence has also been shown in the context of ocular injury, although lineage tracing revealed these cells to be infiltrated monocyte-derived macrophages which were resistant to PLX5622 treatment only in the injured state (Paschalis et al., 2018).

### DAM in glaucoma

Myeloid cells, including resident microglia and recruited monocytes, have gained significant attention in recent years as pathogenic players contributing to RGC loss in glaucoma, the leading cause of irreversible blindness for which elevated intraocular pressure (IOP) is one of the main risk factors (Williams et al., 2017; Zeng and Shi, 2018). These studies date back to early observations of myeloid cell activation and accumulation in the optic nerves of glaucomatous eyes (Neufeld, 1999; Yuan and Neufeld, 2001), which have more recently been shown to include a population of CD163^+^ macrophages (Margeta et al., 2018). Myeloid cell activation has similarly been demonstrated in the DBA/2J mouse model of glaucoma (Bosco et al., 2011) and in other IOP-elevating disease models (Ebneter et al., 2010; Kezic et al., 2013; Bordone et al., 2017), with the observation that immune activation both precedes RGC loss (Bosco et al., 2011) and is positively correlated with the extent of subsequent RGC degeneration (Bosco et al., 2015). These activated myeloid cells have further been shown to up-regulate Toll-like receptor 4 (Tlr4) in glaucoma (Luo et al., 2010), suggesting a role for NF-κB-mediated inflammation in disease pathogenesis. Though the issue of microglial vs. monocyte involvement in glaucoma remains tangled, these studies and others have demonstrated an unequivocal role for neuroinflammatory processes in glaucoma pathogenesis.

In glaucomatous eyes, activated myeloid cells were present in the optic nerve head and co-localized with activation markers and inflammatory mediators, such as CD68 and TNF-α (Neufeld, 1999; Yuan and Neufeld, 2001). Interestingly, genetic deletion of CD11b, a cell-surface integrin expressed by activated myeloid cells, or Tnf-α, a powerful proinflammatory effector critical for macrophage function (Parameswaran and Patial, 2010), has been shown to ameliorate RGC loss in mouse models of glaucoma (Nakazawa et al., 2006). Pharmacological modulation of the immune system has also been shown to prevent disease progression, such that administration ibudilast, a Tlr4 antagonist, or Etanercept, a Tnf-α antagonist, ameliorated experimental RGC loss despite IOP elevation (Roh et al., 2012; Cueva Vargas et al., 2016). Conversely, genetic deletion of the microglial homeostatic checkpoint, *Cx3cr1*, exacerbated both RGC loss and axonal transport dysfunction in glaucoma models (Wang et al., 2014; Breen et al., 2016), perhaps by decreasing the threshold for microglial activation (Yu et al., 2020). Under physiological conditions, Cx3cr1 inhibits the expression of the proinflammatory cytokine Il-1β and monocyte-chemoattractant Ccl2 (Cardona et al., 2006; Combadiere et al., 2007; Sennlaub et al., 2013), suggesting that its down-regulation in the DAM signature might prime the onset of a proinflammatory response propagated by positive feedback mechanisms.

The importance of microglial signaling in glaucoma pathogenesis is supported by our recent work, which revealed a critical role for the DAM signature in the development of glaucoma and progression of RGC degeneration (Margeta et al., 2022). Using bulk RNA sequencing of isolated microglia from two distinct models of glaucomatous degeneration, a DAM was identified which overlapped significantly with the transcriptional profile described in several models of brain neurodegeneration (Keren-Shaul et al., 2017; Krasemann et al., 2017). This signature was characterized by up-regulation of secreted molecules such as *ApoE*, *Lgals3*, proinflammatory cytokines (e.g., *Tnf-*α, *Il-1*β), complement (e.g., *C4b*), and potent chemotaxis molecules (e.g., *Ccl2*, *Ccl12*). As reported by Krasemann et al. (2017) this disease-associated “switch” was controlled by ApoE signaling, such that genetic targeting of *ApoE* prevented acquisition of the DAM profile in glaucoma. Furthermore, selective targeting of ApoE in long-lived myeloid cells was shown to preserve RGCs both structurally and functionally, suggesting that ApoE acts in microglia [and possibly, border-associated macrophages (BAMs)] to promote the onset of neuroinflammation in glaucoma.

We further demonstrated that intravitreal injection of apoptotic neurons was sufficient to induce retinal microglial activation *in vivo*, pointing toward neuronal apoptosis as a critical stimulus for the induction of the DAM profile (Margeta et al., 2022). Furthermore, it was demonstrated that intravitreal injection of phagocytic microglia from donor mice was sufficient to induce RGC loss in the absence of elevated IOP, although the transplanted microglia remained localized in the vitreous cavity. These findings point toward secreted factors as key drivers of microglial cytotoxicity in glaucoma. Indeed, one of the most highly up-regulated molecules downstream of ApoE signaling in glaucoma was Galectin-3, a secreted carbohydrate binding lectin previously implicated in a myriad of CNS degenerations (Jiang et al., 2009; Boza-Serrano et al., 2019; Siew et al., 2019). Galectin-3 deficiency was shown to be neuroprotective in glaucoma, such that genetic or pharmacologic targeting of Galectin-3 conferred robust protection of RGCs despite IOP elevation. Although the mechanism of Galectin-3 cytotoxicity to RGCs remains poorly understood, this molecule is a ligand for Tlr4 (Burguillos et al., 2015; Boza-Serrano et al., 2019), suggesting that it may be upstream of inflammasome-mediated pathways. Furthermore, Galectin-3 binds the phagocytic receptor MerTK (Caberoy et al., 2012), and has been proposed to serve as a “bridge-ligand” linking microglial MerTK to its target cargo (Karlsson et al., 2009; Puigdellivol et al., 2020). Taken together, these studies support a role for Galectin-3 in pathological inflammation and efferocytosis of neurons in glaucoma.

Although mice possess one variant of ApoE, in humans, it is found in three major isoforms – *APOE2*, *APOE3*, and *APOE4* (Farrer et al., 1997), with *APOE4* being well-established as the major risk factor for AD (Corder et al., 1993). One of the key findings from Margeta et al. (2022) is that APOE regulates the DAM signature in glaucoma in an isoform-dependent manner, such that humanized mice carrying the *APOE4* allele exhibit impaired response to neurodegeneration in a manner similar to ApoE KO. Importantly, *APOE4* microglia strongly suppressed proinflammatory mediators such as *Lgals3*, *Tnf-*α, and *Ccl2* despite IOP elevation, while maintaining expression of homeostatic genes such as *Cx3cr1* and *Csf1r*. These results may provide mechanistic understanding for the observed association between the *APOE4* allele and decreased risk of glaucoma in the human population (Mabuchi et al., 2005; Lam et al., 2006; Margeta et al., 2020). Interestingly, the observed microglial quiescence in *APOE4* carriers also supports findings in the field of photoreceptor degeneration, in which subretinal space (SRS) inflammation was reduced in humanized *APOE4* mice compared to *APOE2* and *APOE3* animals (Levy et al., 2015). Although the mechanisms by which APOE isoforms modulate inflammation are poorly understood, it has been shown that APOE4 exhibits severely diminished lipid transport ability compared to its counterparts (Heeren et al., 2004), a functional defect which leads to dysregulated lipid flux in microglia as well as accumulation of intracellular and extracellular cholesterol (Heeren et al., 2004; Sienski et al., 2021; Victor et al., 2022). Future work may examine the effect of APOE variants on cholesterol-associated signaling pathways, including membrane lipid rafts (Chen et al., 2008; Grassi et al., 2020; Lee et al., 2021), as well as the relationship between these pathways and acquisition of the DAM signature.

### DAM in photoreceptor degeneration

Photoreceptor degeneration is a complex neurodegenerative blinding condition with diverse underlying pathologies, including age-related macular degeneration (AMD), retinitis pigmentosa, and other retinal dystrophies, which all converge on the degeneration of rods and cones (Hartong et al., 2006; Wright et al., 2010). The role of microglia and recruited macrophages in photoreceptor degeneration has been an area of investigation since the identification of macrophage accumulation in the interphotoreceptor space of degenerating retinas (Essner and Gorrin, 1979), and later, the discovery that these macrophages contained phagocytosed rhodopsin components (Gupta et al., 2003; Zhao et al., 2015). Retinal microglia predominately reside in two distinct niches in the inner plexiform and outer plexiform layers of the retina, with small numbers in the NFL and GCL, and are absent from the immunosuppressive SRS (O’Koren et al., 2019), which is instead maintained by the phagocytic activity of retinal pigment epithelium (RPE) cells (Ajami et al., 2007; Yu et al., 2020; Figure 1). However, in the context of photoreceptor degeneration, microglia breach the outer retina (Silverman and Wong, 2018; O’Koren et al., 2019; Yu et al., 2020), a response which may be accompanied by infiltration/recruitment of CCR2^+^ monocytes from the blood (Combadiere et al., 2007; Guo et al., 2012; Sennlaub et al., 2013; Zhao et al., 2015; Karlen et al., 2018; Yu et al., 2020). Interestingly, compared to engrafted macrophages, adult retinal microglia do not up-regulate the classic DAM marker Cd11c in response to certain models of photoreceptor degeneration (O’Koren et al., 2016). However, this deficiency appears to be selective, as other DAM markers (e.g., *Lgals3*, *ApoE*, *LpL*, *Spp1*, *Gpnmb*, and *Fabp5*) remain significantly up-regulated in this disease context (O’Koren et al., 2019).

Microglia and macrophages contribute to tissue repair throughout the body but are subsequently eliminated to allow for resolution of inflammation (Buckley et al., 2013; Gautier et al., 2013); however, in the case of uncontrolled photoreceptor degeneration, their presence in the SRS becomes chronic and associated with secretion of proinflammatory cytokines, including Tnf-α and Il-1β (Yoshida et al., 2013; Appelbaum et al., 2017). Infiltrating monocytes are actively recruited by resident macrophages by Ccl2-Ccr2 chemokine attraction, a mechanism which may drive local proinflammatory cascades as blood-derived monocytes down-regulate their Ccr2 expression and differentiate into macrophages with high expression of Tnf-α, Il-1β, Il-6, and Ccl2, as well as profibrotic and angiogenic factors (Wynn et al., 2013; Yu et al., 2020). Certain studies have pointed toward a critical role for monocyte infiltration in photoreceptor degeneration, with Ccr2 blockade resulting in complete neuroprotection in a *Cx3cr1*-deficiency model (Sennlaub et al., 2013). Similar results have been shown in an immunization-induced model of AMD (Cruz-Guilloty et al., 2013) and in the *rd10*^–/–^ model of retinitis pigmentosa (Guo et al., 2012).

Recently, an opposing role for monocytes and microglia has also been demonstrated in the *rd10*^–/–^ model, whereby attenuation of circulating monocyte infiltration decreased cone degeneration, but depletion of resident microglia exacerbated it (Funatsu et al., 2022). This finding is supportive of prior work in the *rd10*^–/–^ model which demonstrated C3-CR3 signaling by Iba1^+^ macrophages as critical for preserving photoreceptor integrity, although this study did not distinguish between yolk sac- and bone marrow-derived lineages (Silverman et al., 2019). Interestingly, the absence of C3 or CR3 in the retinitis pigmentosa model increased macrophage cytotoxicity and decreased physiological clearance of apoptotic photoreceptors. Despite evidence pointing toward a reparative role for microglia, it has been proposed that microglia preferentially phagocytose stressed but viable photoreceptors due to their proximity to photoreceptor cell bodies, active phagocytic extensions and intracellular phagosomes, and actively surveillant behavior compared to engrafted macrophages (Zhao et al., 2015). Additional studies will be needed to definitively resolve this question, and interactions between monocyte-derived macrophages and microglia during photoreceptor degeneration remain an open area of investigation.

Considering the challenge of identifying stable cell-type specific markers for different myeloid cell subpopulations, fate mapping has become the experimental approach of choice for differentiating between resident microglia and blood-derived monocytes (Parkhurst et al., 2013; Yona et al., 2013). Using fate mapping in combination with single cell RNA sequencing, a report by O’Koren et al. (2019) identified a unique transcriptional profile of subretinal microglia during photoreceptor degeneration which is distinct from that of infiltrating monocytes, and which shares significant similarities to the DAM phenotype, including up-regulation of *Lgals3*, *LpL*, *Spp1*, *Trem2*, and *Cd68* (Keren-Shaul et al., 2017; Krasemann et al., 2017). Conditional depletion of microglia prior to light damage or in the *Rho*^P23H/WT^ model of retinal dystrophy aggravated neurodegeneration, indicating a neuroprotective role for the DAM signature in these contexts (O’Koren et al., 2019). Microglia from both the inner and outer plexiform layers migrated to the SRS following light damage; however, the neuroprotective response was shown to be niche-specific, specifically requiring Il-34-dependent microglia from the inner plexiform layer (IPL). In contrast, peripheral macrophages repopulated the neuroretina following microglial depletion but were virtually absent from the SRS in these models (O’Koren et al., 2019).

The report by O’Koren et al. builds upon a wealth of literature which has demonstrated migration of inner retinal microglia and infiltration of macrophages in response to photoreceptor degeneration; however, these studies have predominately pointed toward a pathogenic role for mononuclear phagocytes in the outer retina and SRS (Combadiere et al., 2007; Guo et al., 2012; Cruz-Guilloty et al., 2013; Sennlaub et al., 2013; Levy et al., 2015; Calippe et al., 2017). It is thus interesting to speculate why the DAM phenotype described by O’Koren et al. is neuroprotective, in contrast to what has been described in glaucoma (Margeta et al., 2022). A potential line of reasoning for this discrepancy may include a compensatory role for microglia in promoting photoreceptor integrity and phagocytosing spent outer segment disks, an RPE-mediated maintenance process which may be interrupted in the case of RPE dysfunction (Young and Bok, 1969; Finnemann et al., 1997; Bazan, 2007; Dransfield et al., 2015; Yu et al., 2019; Vargas and Finnemann, 2022). Indeed, photoreceptor degeneration likely involves a complex interplay between photoreceptors, RPE, and infiltrating myeloid cells, a dynamic which is absent from degenerations involving the inner retina, including glaucoma. Alternatively, it is intriguing to note the relationship between neuroprotection and the absence of proximate monocyte-recruits, as the SRS was shown to be a microglia-privileged niche in both the light damage and *Rho*^P23H/WT^ models employed by O’Koren et al. (2019). From these studies and others, a key question emerging in the field is the dynamic interplay between microglia and infiltrating monocytes, as well the pathways which may shift the balance from a net-reparative to net-degenerative microglial response.

### Heterogeneity within the DAM signature

Marker specificity continues to be one of the greatest hurdles in distinguishing the roles of microglia and peripherally derived macrophages in disease. This matter is further complicated by the presence of BAMs, a long-lived myeloid cell population that includes dural, perivascular, and choroid plexus macrophages (Prinz et al., 2021). Though the pan-macrophage nature of markers such as Iba1, CD11b, and Cx3cr1 is historically well established (Amici et al., 2017; Reyes et al., 2017), such considerations have more recently been extended to include a broader suite of cell-surface markers and sorting strategies (Yu et al., 2020). Additionally, monocyte-derived macrophages have been shown to repopulate the brain following microglial depletion and up-regulate a suite of microglial markers, including *P2ry12*, *Tmem119*, and *Fcrls*, suggesting that these markers may be niche-specific rather than lineage-specific in the CNS (Bennett et al., 2018; Lund et al., 2018b). A similar acquisition of microglial surface markers has also been demonstrated by infiltrating macrophages in response to photoreceptor degeneration (O’Koren et al., 2019) and ocular injury (Paschalis et al., 2019; Lei et al., 2021). However, whether the acquisition of these microglial markers by peripherally derived cells implies long-term functional equivalence has not been thoroughly investigated.

Considering persistent overlaps in microglial marker expression by different myeloid cell subpopulations, resolving the ontogeny of the DAM population has been a matter of some debate (Jay et al., 2015; Van Hove et al., 2019; Silvin et al., 2022). To resolve this uncertainty, recent advances in single cell RNA sequencing have enabled generation of an integrated immune map which captures myeloid cell heterogeneity throughout development, aging, and disease with single cell resolution (Silvin et al., 2022). One of the most striking observations taken from this integration was the composition of the area representing developmental microglia. Although this cluster was predominately comprised of cells from embryonic and postnatal periods (Hammond et al., 2019; Silvin et al., 2022), it also contained a significant proportion of cells from AD mice (Keren-Shaul et al., 2017; Van Hove et al., 2019), suggesting that microglia undergo a developmental-like reprogramming in the context of neurodegeneration. This finding builds on prior reports demonstrating significant similarities between DAM microglia in AD and CD11c^+^ microglia in development (Hagemeyer et al., 2017; Wlodarczyk et al., 2017; Anderson et al., 2019a; Li et al., 2019).

It was also discovered that myeloid cells expressing disease-associated markers from the original Keren-Shaul et al. (2017) dataset were localized in two distinct areas on the integrated map: one in the developmental microglia area, which was enriched for Cd11c (*Itgax*), and one in the mature microglia area, which exhibited high expression of proinflammatory mediators (Silvin et al., 2022). The cells whose transcriptional signatures positioned them in the developmental microglia area were thus referred to as DAMs, for *bona fide* DAMs, due to their expression of classical DAM markers including *Itgax* and *Spp1*. These cells displayed anti-inflammatory and pro-phagocytic expression profiles. In contrast, cells localized in the mature microglial area were referred to as DIMs due to their high expression of proinflammatory pathways, including *Il-1*α, *Il-1*β, *Il-6*, *Tnf-*α, and molecules involved in Tlr signaling, as well as increased nitric oxide (NO) and reactive oxygen species (ROS) production. DIMs were shown to be relatively sparse during development but accumulated significantly both in AD and in aging, comprising approximately 25% of isolated microglia from brains of P540 mice (Hammond et al., 2019; Silvin et al., 2022). Importantly, lineage tracing using the Ms4a3-tdTomato fate mapping strategy demonstrated that DIMs were monocyte-derived macrophages, while *bona fide* DAMs were yolk sac-derived microglia (Silvin et al., 2022). The accumulation of DIMs in aging and neurodegenerative disease is in line with prior reports demonstrating compromised blood-brain-barrier integrity in these settings (Rustenhoven and Kipnis, 2019).

**FIGURE 2:**
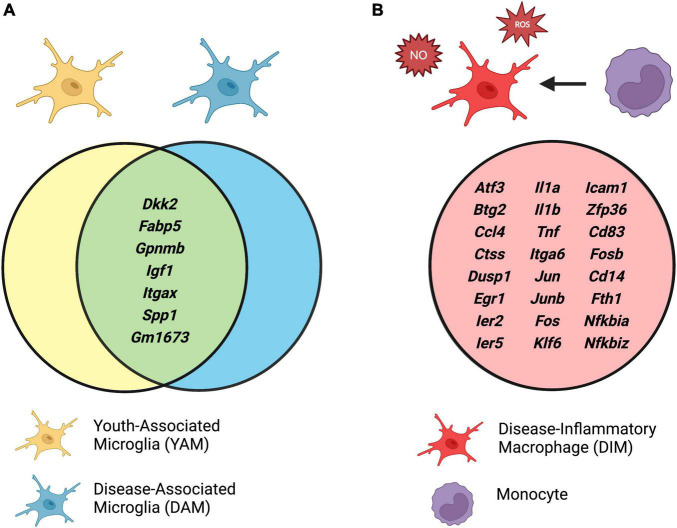
**(A)** Conserved gene expression between disease-associated microglia (DAMs) and youth-associated microglia (YAMs), and **(B)** unique expression profile of disease-inflammatory macrophages (DIMs). Identified by integrated single cell RNA sequencing analyses of isolated brain myeloid cells in mouse models of Alzheimer’s disease (AD) and aging. Adapted from Silvin et al. (2022). Created with www.biorender.com.

A comparison of mRNA transcript enrichment revealed that both DIMs and DAMs express the homeostatic microglial markers *P2ry12* and *Cx3cr1*; however, these markers were relatively higher in DIMs (Silvin et al., 2022). Similarly, both DAMs and DIMs expressed *Trem2*, although only DAMs were Trem2-dependent. Differential gene expression analysis of DAMs and DIMs revealed distinct transcriptional signatures conserved by these populations in both murine and human single cell RNA sequencing datasets (Figure 2). Several of these core DAM genes were also conserved in CD11c^+^ developmental microglia [termed youth-associated microglia (YAMs) by Silvin et al. (2022)], including *Itgax*, *Igf1*, *Spp1*, *Gpnmb*, and *Dkk2*. Furthermore, comparative pathway analysis of the DAM and YAM populations demonstrated that DAMs were enriched for anti-inflammatory functions, reflecting their distinct role in response to disease. It is interesting to note that the *bona fide* DAM population was critically dependent on Trem2, such that these cells were completely absent in Trem2 KO mice and critically reduced in AD-Trem2 KO mice (Keren-Shaul et al., 2017; Silvin et al., 2022). Conversely, the inflammatory DIM population expanded dramatically in the absence of Trem2 both at baseline and in AD, a trend which seems to mirror the accumulation of Trem2-independent Stage 1 DAM in the original report (Keren-Shaul et al., 2017). Taken together, these single cell RNA sequencing analyses point toward an anti-inflammatory role for Trem2 signaling in the brain.

Although the DAM-DIM dichotomy has thus far been described only for brain microglia, it is tempting to speculate that similar heterogeneity may exist in the diseased retina, suggesting that monocyte-derived macrophages may play a critical role in pathogenesis of retinal neurodegeneration.

## Discussion and perspectives

Microglia are resident immune cells of neural tissue, with a myriad of functions in development, adulthood, aging, and disease (Butovsky and Weiner, 2018; Li and Barres, 2018). One of the most interesting facets of microglial biology is the emergence of a DAM signature in neurodegenerative disease, also referred to as the MGnD (Keren-Shaul et al., 2017; Krasemann et al., 2017). Although this signature is broadly defined, its intersection with developmental microglial signatures (i.e., *bona fide* DAM) reflects an up-regulation of phagocytic, metabolic, and anti-inflammatory pathways required to maintain tissue homeostasis during periods of substantial tissue remodeling (Silvin et al., 2022). In development, this signature has been shown to mediate both cell elimination and cell survival and to promote physiological synaptic pruning and white matter refinement (Hagemeyer et al., 2017; Wlodarczyk et al., 2017; Anderson et al., 2019a; Li et al., 2019). Prior work has also pointed toward a physiological role for Trem2-dependent pathways in disease (Keren-Shaul et al., 2017; Condello et al., 2018; Gratuze et al., 2018), although the net effect of the DAM signature remains a matter of debate. In the light damage model of photoreceptor degeneration, microglia up-regulate Trem2-dependent and *bona fide* DAM genes including *LpL*, *Spp1*, *Gpnmb*, *Fabp5*, and *Cd68* and are neuroprotective in this context (O’Koren et al., 2019; Yu et al., 2020). Considering the multifaceted nature of the DAM signature, a significant parsing of its component pathways may enable new insights into microglial biology in development and disease.

Across the organismal lifespan, neuronal apoptosis has emerged as a common stimulus leading to DAM activation. Prior reports have demonstrated that the presence of neuronal apoptosis may contribute to erroneous clearance of still viable neurons (Zhao et al., 2015); however, cell death in itself should not be seen as an inherently pathogenic factor considering the vast proportions of CNS cells which must be cleared and metabolized in the context of neurodevelopment. Thus, it is possible that microglial clearance of neurons becomes pathological in disease due to interactions with other cell types that are absent in development, including monocyte-derived DIMs. In this review, we note two instances where the role of DAM is cited to be beneficial rather than detrimental: in the case of development and in the light damage and *Rho*^P23H/WT^ mouse models of photoreceptor degeneration (Anderson et al., 2019a,b, 2022; O’Koren et al., 2019). Notably, these studies reflect time-points and conditions in which DAM microglia are operating in a microglia-privileged niche, without significant contributions from blood-derived monocytes. In contrast, it is possible that the deleterious role for the DAM signature in glaucoma represents pathogenic interactions between microglia and recruited monocytes, whereby ApoE expression in long-lived resident myeloid cells is necessary to initiate the inflammatory response (Margeta et al., 2022) but recruited monocyte-derived macrophages play a key pathogenic role (Howell et al., 2012; Williams et al., 2019; Chen et al., 2020). Interestingly, the detrimental effect of monocyte-derived macrophages may be regulated by TGF-β, as it has been shown that TGF-β-deficient monocytes drive fatal demyelinating disease following engraftment in the spinal cord, with strong up-regulation of disease-associated molecules including *Lgals3* (Lund et al., 2018a). Future studies may serve to elucidate the functional role of engrafted macrophages in neurodegenerative contexts as well as their long-term interactions with resident microglia.

In contrast to the anti-inflammatory profile of *bona fide* DAMs, the proinflammatory nature of DIMs suggests that the infiltration of these cells to parenchyma may create vicious positive feedback cycles, such as those underpinning glaucoma and photoreceptor degeneration (Alqawlaq et al., 2019; Yu et al., 2020). There is a strong correlation between DIM-conserved markers in the brain and the proinflammatory molecules known to be cytotoxic in retinal degeneration, including Il-1α, Il-1β, Tnf-α, and Tlr4, as well as NO and ROS production (Silverman and Wong, 2018; Alqawlaq et al., 2019; Wooff et al., 2019; Yu et al., 2020; Baudouin et al., 2021; Coyle et al., 2021). Thus, it could be proposed that the primary source of these molecules in the degenerating retina may be monocyte-derived macrophages, which up-regulate a suite of microglial markers following engraftment and thus become difficult to distinguish based on marker expression alone (Bennett et al., 2018; Lund et al., 2018b; Paschalis et al., 2019; Lei et al., 2021). Consistent with this idea, monocytes have been shown to infiltrate the retina following ocular injury and cause RGC loss *via* secretion of proinflammatory cytokines, including Tnf-α and Il-1β, which remained chronically up-regulated by engrafted macrophages despite differentiation into quiescent microglial morphology (Paschalis et al., 2018; Chen et al., 2020). Similarly, in models of epilepsy, it has been demonstrated that Tnf-α and Il-1β levels are hundreds fold higher in circulating monocytes compared to microglia at baseline; however, while microglia up-regulated these markers in response to insult, their levels in monocytes remained stably high after brain entry (Varvel et al., 2016). Although additional lineage tracing studies are needed, these findings suggest that microglia are dynamic responders to injury, while engrafted macrophages exert constant inflammatory influence on the CNS. A similar perspective has been highlighted in a recent article on the role of CNS mononuclear phagocytes in health and disease, which proposes that tissue-resident macrophages (i.e., microglia and BAMs) exert tissue-protective functions such as debris clearance and functional support, while blood-borne phagocytes are the primary drivers of neuroinflammation (Mundt et al., 2022).

Although infiltrating monocytes appear to act in detrimental fashion upon recruitment to the retina and optic nerve, an open question remains if such a response is inevitably maladaptive. It could be proposed that monocyte infiltration to the site of CNS injury reflects an inappropriate extension of their response to peripheral nerve damage, where their action is an adaptive process leading to the clearance of growth-inhibitory myelin debris and functional nerve repair (Nguyen et al., 2002; Barrette et al., 2008; Parrinello et al., 2010; Cattin et al., 2015). Following damage to peripheral nerves, it has been shown that hypoxic conditions induce recruitment of vascular endothelial growth factor (VEGF)-expressing macrophages, which guide the reparative action of Schwann cells by promoting local angiogenesis (Cattin et al., 2015). In the CNS, studies aiming to promote the regenerative capacity of the optic nerve have shown that inflammatory monocyte-derived factors are critical for promoting RGC axon regeneration following ONC (Leon et al., 2000; Yin et al., 2003; Peterson et al., 2021; Xie et al., 2022), although to date the extent of this recovery remains limited. The beneficial contribution of macrophages to acute CNS injury is further complicated by their concurrent secretion of molecules with deleterious effects on neurons, indicating that their presence in injured neural tissue is a double-edged sword (Yin et al., 2003). Taken together, it is possible that immune mechanisms which successfully promote regeneration of peripheral nerves become maladaptive in the case of chronic CNS neurodegeneration, due in part to prolonged production of proinflammatory cytokines and cytotoxic agents. Future work may investigate the mechanisms by which monocytes enter the retina and interact with resident microglia, as these pathways could represent promising therapeutic targets for a range of chronic neurodegenerative diseases of the eye.

## Conclusion

Microglial transcriptional signatures are the result of complex interactions between these cells and the CNS microenvironment (Gosselin et al., 2017). Although various acronyms have gained widespread use as categorization tools used to characterize microglial phenotypes and functions, new perspectives in the field have emphasized that microglial states are not binary switches, but rather complex transcriptional landscapes existing along continuums (Paolicelli et al., 2022). These transcriptional states may reflect extrinsic properties such as life stage, CNS region, sex, and disease status (Paolicelli et al., 2022) but also integrate cell-intrinsic properties that have yet to be elucidated (Stratoulias et al., 2019). In the context of neurodegeneration, microglia acquire a molecular profile characterized by up-regulation of phagocytic and metabolic machinery that is shared by microglia in various developmental contexts. Although a simplification of complex biology, the intersection of these transcriptional signatures suggests the existence of recycled gene programs which play a role both in the early sculpting of neural circuits as well as in response to aging and disease (Keren-Shaul et al., 2017; Silvin et al., 2022).

Despite marked overlap with developmental microglial signatures, prior studies have demonstrated that DAM microglia are a heterogenous population associated with both anti-inflammatory and proinflammatory properties (Keren-Shaul et al., 2017; Krasemann et al., 2017). Reflecting this complexity, it has been shown that resident microglia play a deleterious role in glaucoma (Margeta et al., 2022) but are beneficial in certain models of photoreceptor degeneration in which there is minimal recruitment of monocyte-derived macrophages to the site of injury (O’Koren et al., 2019). It therefore appears plausible that it is the crosstalk between resident microglia and infiltrating monocytes in disease which tips the scales to a net-degenerative outcome by facilitating an exaggerated immune response. Indeed, monocytes are remarkably plastic, and become difficult to distinguish from microglia following CNS infiltration by marker expression alone (Bennett et al., 2018; Lund et al., 2018b; Paschalis et al., 2019; Lei et al., 2021). Our understanding of microglial and macrophage phenotypes in disease is rapidly evolving, and it remains possible that the DAM signature reflects dynamic neuroimmune interactions which include context-dependent contributions from peripheral macrophages.

The notion that engrafted macrophages may be masquerading as microglia while actively contributing to neurodegenerative disease pathology represents a controversial topic in the field; nonetheless, it may be one warranting additional scrutiny as lineage tracing and single cell RNA sequencing technologies enable more granular investigations of macrophage phenotypes and functions. Important topics to address in future work include the infiltration, proliferation, and lifespan of monocyte-derived cells in the degenerating retina, as well as the interactions between these cells and resident microglia in disease initiation and propagation.

## Author contributions

KP: original draft. MM and KP: revisions and editing. Both authors contributed to the manuscript in conceptualization and approved the submitted version.
